# Activation of Innate and Adaptive Immunity in the Central Nervous System Following Xenogeneic Mesenchymal Stromal Cell Transplantation

**DOI:** 10.3390/cells15211966

**Published:** 2026-10-28

**Authors:** María Norte-Muñoz, Joaquín Cantón-Sandoval, Fernando Lucas-Ruiz, Victoriano Mulero, Marta Agudo-Barriuso, David García-Bernal

**Affiliations:** 1 Experimental Ophthalmology Research Group, Department of Ophthalmology, Optometry, Otorhinolaryngology, and Pathological Anatomy, Faculty of Optics and Optometry, University of Murcia, 30100 Murcia, Spain; maria.norte@um.es (M.N.-M.); fernando.lucas@um.es (F.L.-R.); 2 Instituto Murciano de Investigación Biosanitaria Pascual Parrilla (IMIB), 30120 Murcia, Spain; vmulero@um.es; 3 Department of Developmental and Molecular Biology, Albert Einstein College of Medicine, New York, NY 10461, USA; joaquincs96@gmail.com; 4 Immunity, Inflammation, and Cancer Research Group, Department of Cell Biology and Histology, Faculty of Biology, University of Murcia, 30100 Murcia, Spain; 5 Hematopoietic Transplantation and Cellular Therapy Research Group, Department of Biochemistry, Molecular Biology and Immunology, Faculty of Medicine, University of Murcia, 30120 Murcia, Spain

**Keywords:** stem cell transplantation, mesenchymal stromal cells, immune response, central nervous system, innate and adaptive immunity

## Abstract

**Highlights:**

**What are the main findings?**

- Xenogeneic intravitreal transplantation of hBM-MSCs in mice induces strong local retinal immune activation, characterized by resident microglial activation and leukocyte infiltration.
- Both cellular and humoral immune responses are observed, involving T cells and NK cells, together with the production of IgG antibodies against human antigens.

**What are the implications of the main findings?**

- Retinal immune privilege is insufficient to prevent immune rejection of xenogeneic cell grafts.
- Human-cell xenotransplantation models have important translational limitations and should be complemented by more clinically relevant allogeneic or syngeneic transplantation settings.

**Abstract:**

Stem cell-based therapies have emerged as promising strategies for neurodegenerative disorders, partly supported by the concept of central nervous system immune privilege, which has encouraged xenogeneic transplantation approaches under the assumption of limited host immune responses. However, the immunological consequences of xenogeneic cell delivery into the central nervous system remain poorly characterized. In this study, we investigated the immune response elicited by intravitreal transplantation of human bone marrow-derived mesenchymal stromal cells (BM-MSCs) into healthy mouse retinas. Twenty-one days after transplantation, retinal CD45^+^ and/or CD11b^+^ cells, including microglia, macrophages, and infiltrating leukocytes, were isolated and subjected to exploratory single-cell RNA sequencing. Candidate immune cell populations and pathways identified transcriptomically were subsequently evaluated by immunostaining, flow cytometry, and plasma antibody analyses. Xenogeneic BM-MSC transplantation induced marked transcriptional changes consistent with immune activation, enhanced antigen presentation, and leukocyte recruitment, features that are not typically associated with central nervous system homeostasis. These findings were supported by complementary cellular and systemic analyses, collectively revealing innate and adaptive immune responses directed against the transplanted human cells. The results demonstrate that xenogeneic BM-MSC transplantation can overcome local immune regulatory mechanisms and trigger robust immune activation within the healthy retina. These findings identify previously underappreciated immunological barriers to xenogeneic stem cell-based therapies and reveal coordinated innate and adaptive immune responses despite the presumed immune privilege of the CNS. Consideration of these immune responses will be essential for the safe and effective translation of regenerative therapies.

## 1. Introduction

Preclinical cell therapy studies predominantly assess the behaviour of human cells in rodent disease models [1]. In systemic disease models, animals are routinely immunosuppressed to prevent xenogeneic immune rejection. In contrast, studies targeting the central nervous system (CNS) often omit immunosuppression, reflecting the long-standing assumption of CNS immune privilege [2,3,4,5,6]. This assumption has contributed to an underestimation of the immunological risks associated with xenogeneic cell transplantation into the CNS. Because graft rejection is not anticipated, local and systemic immune responses, as well as the potential effects of transplanted cells on healthy CNS tissue, are rarely examined. However, accumulating evidence indicates that the CNS can mount substantial immune responses under specific conditions, particularly following injury or therapeutic intervention [7,8,9]. These findings challenge the traditional view of CNS immune privilege and underscore the need to reassess the immunological safety of cell-based therapies approaching clinical translation for neurodegenerative diseases such as glaucoma, Parkinson’s disease, and multiple sclerosis [10]. In the present study, the retina was used as a model of the CNS because it develops from the neural tube and shares key structural, cellular, and immunological features with other CNS regions, while offering unique accessibility for in vivo transplantation and immune-response analyses [11,12].

Cell-based therapies for the neurodegenerating CNS aim not only to replace lost neurons [13] but also to preserve or restore neuronal function through neuroprotective mechanisms [14,15,16,17]. Among the various cell types investigated, mesenchymal stromal cells (MSCs) are among the most extensively characterized. MSCs offer practical advantages, including accessibility from adult tissues, and exhibit functional properties such as immunomodulatory activity, secretion of trophic factors, and the capacity to dynamically adjust their secretome in response to the surrounding microenvironment [18,19,20]. These features have positioned MSCs as promising candidates for neuroprotective applications [21].

Using the rodent retina as a CNS model, our previous studies employing bone marrow-derived MSCs (BM-MSCs) demonstrated that therapeutic efficacy is critically influenced by the transplantation modality [15,17,22]. In transplantation biology, syngeneic transplantation refers to donor and recipient individuals that are genetically identical, allogeneic transplantation involves individuals of the same species but with different genetic backgrounds, and xenogeneic transplantation involves donor and recipient from different species. Transplanted BM-MSCs do not survive long term due to host immune rejection and are consistently observed surrounded by activated microglia, highlighting the detrimental impact of immune-mediated inflammation on therapeutic outcomes. Notably, syngeneic BM-MSC transplantation yields superior neuroprotective effects compared with allogeneic or xenogeneic approaches, a difference that correlates with reduced glial activation. In contrast, xenotransplantation induces stronger activation of both microglia and macroglia, accompanied by increased expression of proinflammatory mediators [23,24]. Moreover, leukocyte infiltration into the retinal parenchyma has been observed exclusively after xenogeneic transplantation, suggesting immune activation beyond innate mechanisms and raising the possibility of adaptive or humoral immune involvement. Nevertheless, the nature and extent of the immune response triggered by xenogeneic MSC transplantation remains incompletely understood. Despite the widespread use of this approach in preclinical CNS research, the immune cell populations and pathways potentially involved have not been explored at single-cell resolution [25].

Although humoral immune responses have been described following intravitreal administration of viral vectors in rodents and humans [26], comparable responses have not been previously characterized in the context of cell-based therapies. Therefore, the aim of this study was to characterize the nature and magnitude of the immune response elicited by xenogeneic BM-MSC transplantation into the healthy CNS and to identify the immune-cell populations and pathways potentially involved. To address this objective, CD45^+^ and/or CD11b^+^ cells were isolated from intact mouse retinas and from retinas that had received intravitreal human BM-MSC transplantation 21 days earlier, following established protocols [23]. These populations, which likely included microglia, macrophages, and infiltrating leukocytes [27], were subjected to exploratory single-cell transcriptomic profiling to identify candidate immune-cell populations and pathways potentially involved in the response. The resulting observations were subsequently examined using complementary immunodetection, flow cytometry, and plasma antibody analyses, revealing a humoral immune response consistent with recognition of the xenotransplanted human cells, with important implications for cell-based CNS therapies.

## 2. Materials and Methods

### 2.1. Study Aims and Design

**Figure 1:**
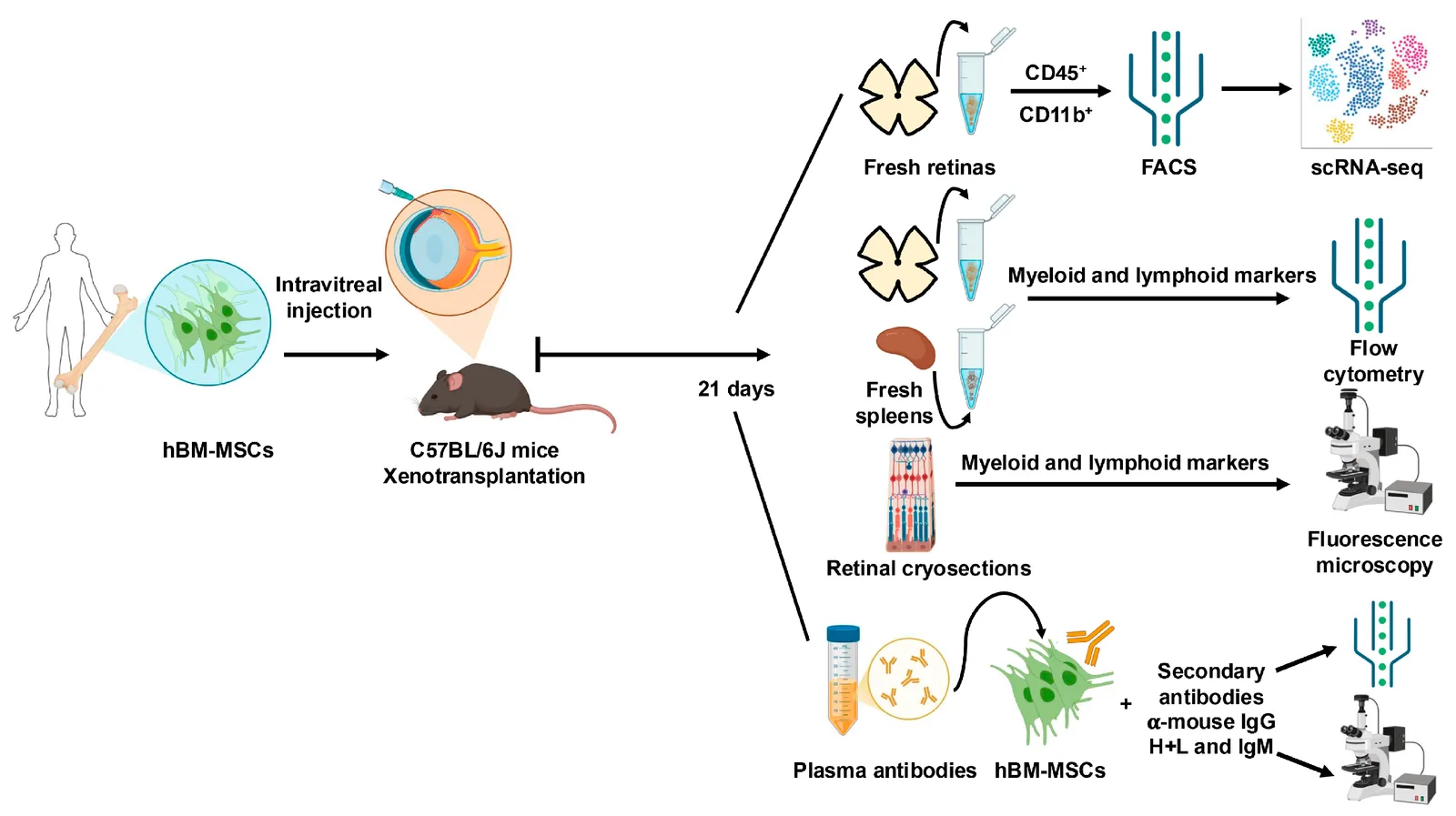
Experimental design. Experimental analyses were performed 21 days after intravitreal injection of hBM-MSCs into the left eyes of C57BL/6J mice. Fresh retinas were processed for scRNA-seq and flow cytometry to characterize immune cell populations, with spleen samples included in cytometric assays. Fixed retinas were analysed by immunodetection to evaluate immune cell infiltration. Serum from xenotransplanted animals were used to detect antibodies against hBM-MSCs, employing both immunodetection on fixed cells and by flow cytometry following cell incubation. Sample size was *n* = 4–8 per assay. Images were created with NIH Bioart source (https://bioart.niaid.nih.gov/, accessed on 19 January 2026). Abbreviations: hBM-MSCs: human bone marrow mesenchymal stromal cells; scRNA-seq: single-cell RNA sequencing; FACS: fluorescence-activated cell sorting.

This study aimed to characterize the immune response elicited by xenogeneic stem cell transplantation into the healthy CNS, using the retina as a model. Human BM-MSCs from healthy donors were injected intravitreally into C57BL/6J mice (*n* = 22). Twenty-one days post-transplantation, retinas were harvested and processed either as fresh tissue for molecular analyses (scRNA-seq and flow cytometry) or as perfused tissue for histological evaluation (immunofluorescence on cryosections). Flow cytometry was also performed on spleens to assess systemic adaptive immunity. Serum samples were analyzed for the presence of mouse antibodies directed against xenotrasplanted cells using both flow cytometry and immunodetection assays. Intact animals were used as controls (*n* = 18) or as BM-MSC donors (*n* = 10). This multimodal approach enabled the identification of both innate and adaptive immune responses. Single-cell transcriptomic profiling was used as an exploratory discovery approach, and the resulting observations were subsequently evaluated using independent cellular and humoral immune assays. A graphical overview of all experimental procedures is depicted in Figure 1.

### 2.2. Animals

All animal procedures were approved by the Institutional Animal Care and Use Committee of the University of Murcia (Murcia, Spain) and performed according to our institutional guidelines (approved protocol A1320140704) and ARRIVE guidelines.

Two-months-old male C57BL/6J mice were obtained from the breeding colony at the University of Murcia (total number of animals used in this study = 50). No animals were excluded from this work. Animals were randomly allocated to experimental or control groups. Sample size was decided based on our previous expertise with this model [15,23]. Experiments, assessment, and data analyses were not blinded.

The animals were housed in the animal facility of the University of Murcia under controlled environmental conditions (temperature- and light-regulated rooms; 12 h light/dark cycles) with food and water provided ad libitum.

Intravitreal injections were conducted under general anaesthesia induced via intraperitoneal administration of a ketamine (60 mg/kg; Ketolar, Parke-Davies S.L., Barcelona, Spain) and xylazine (10 mg/kg; Rompún, Bayer S.A., Barcelona, Spain) mixture. Anaesthesia was confirmed by the absence of the pedal withdrawal reflex and by monitoring respiratory rate and lack of response to surgical manipulation. Animals were maintained on a heated pad throughout the procedure to preserve physiological temperature. Analgesia was provided by subcutaneous administration of buprenorphine (0.1 mg/kg; Buprex, Buprenorphine 0.3 mg/mL; Schering-Plough, Madrid, Spain) 15 min before the procedure. To prevent corneal desiccation, a lubricating ophthalmic ointment (Tobrex; Alcon S.A., Barcelona, Spain) was applied to the eyes during and after anesthesia. Following completion of all procedures, animals were euthanized under deep anaesthesia by intraperitoneal injection of an overdose of sodium pentobarbital (200 mg/kg; Dolethal, Vetoquinol; Especialidades Veterinarias S.A., Alcobendas, Madrid, Spain). Death was confirmed by cessation of respiration, lack of heartbeat, and absence of corneal and pedal reflexes, in accordance with current European and institutional ethical guidelines.

### 2.3. Isolation and Culture of Human and Mouse Bone Marrow Mesenchymal Stromal Cells (hBM-MSCs and mBM-MSCs)

Human bone marrow samples were collected through direct percutaneous aspiration from the iliac crest of six healthy volunteers at the Virgen de la Arrixaca University Clinical Hospital (Murcia, Spain). All procedures for human sample collection and processing received approval from the local ethics committees (HCUVA10/13-25/11/2013), in accordance with the Spanish and European legislation and conformed to the ethical guidelines of the Helsinki Declaration. Donors provided written informed consent before obtaining samples. Bone marrow was collected in syringes containing 20 U/mL sodium heparin (ROVI Pharmaceutical Laboratories, Madrid, Spain). Mononuclear cells were isolated by Ficoll-Paque density gradient centrifugation, as previously described [28].

Subsequently, the mononuclear fraction was washed twice with phosphate-buffered saline (PBS; Merck Life Science, Madrid, Spain) and plated in 75-cm^2^ culture flasks (Nunc, Roskilde, Denmark) at a density of 1.6 × 10^5^ cells/cm^2^ in Minimum Essential Medium Eagle (Thermo Fisher Scientific, Madrid, Spain) supplemented with 10% fetal bovine serum (FBS; HycloneTM; Thermo Fisher Scientific), 1% penicillin/streptomycin (P/S; Thermo Fisher Scientific, Madrid, Spain), and 1% GlutaMAX™ (Thermo Fisher Scientific, Madrid, Spain). After three days of incubation at 37 °C in 5% CO_2_, non-adherent cells were removed, and fresh medium was added, with medium changes twice weekly.

Human BM-MSCs were characterized by their immunophenotype (CD73^+^, CD90^+^, CD105^+^, CD14^−^, CD20^−^, CD34^−^ and CD45^−^) and trilineage differentiation capacity (adipogenic, osteogenic and chondrogenic differentiation). In addition, their immunomodulatory properties were verified through inhibition of activated human T-cell proliferation. Representative characterization data are provided in Supplementary Figure S1A,B, and detailed methodologies have been described previously [23].

Mouse bone marrow mesenchymal stromal cells (mBM-MSCs) were isolated from the femurs and tibias of 6–8-week-old C57BL/6 mice (*n* = 10). The culture protocol was similar to that used for human samples, as previously described [23]. These cells were used as a control to assess the specificity of the immune antibody responses against human antigens.

### 2.4. Intravitreal Injections

Human BM-MSCs were resuspended in DMEM medium at a concentration of 8 × 10^3^ cells/µL. A dose of 2 × 10^4^ hBM-MSCs per eye in a total volume of 2.5 µL was administered intravitreally into the left eye, following previously established protocols [23,29,30].

### 2.5. Fluorescence-Activated Cell Sorting (FACS) and Single-Cell RNA Sequencing (scRNA-Seq)

Following euthanasia, fresh retinas (*n* = 4 control, *n* = 4 xenotransplanted) were collected and placed in Neurobasal medium (Thermo Fisher Scientific, Madrid, Spain), supplemented with 10% FBS, 2% B-27 supplement (Sigma-Aldrich, St. Louis, MO, USA) and 1% GlutaMAX™. The retinas were then mechanically dissected and subjected to enzymatic digestion using 0.2% collagenase-A (0.223 U/mg; Roche Diagnostics, Basel, Switzerland) in DMEM medium. Samples were incubated at 37 °C for 30 min, after which the resulting cell suspensions were filtered through a 70-µm cell strainer (Corning™, Thermo Fisher Scientific, Madrid, Spain) and centrifuged at 600 *g* for 5 min. Following digestion, cell viability was determined by trypan blue exclusion, yielding consistently high viability rates (≥85%).

The cell pellet was resuspended in DMEM medium and stained with the following fluorochrome-labelled primary antibodies, both from eBiosciences (San Diego, CA, USA): anti-mouse CD11b-FITC (clone M1/70) at a dilution of 1:250, and anti-mouse CD45-PE (clone 30-F11) at a dilution of 1:500. After 30 min of incubation at 4 °C, the cells were washed twice, centrifuged, and resuspended in Neurobasal medium supplemented with 2% bovine serum albumin (BSA; Thermo Fisher Scientific, Madrid, Spain). The cell suspensions were then immediately sorted using a SH800Z flow cytometer cell sorter (Sony Biotechnology Inc., San Jose, CA, USA) and the CD45^+^ and/or CD11b^+^ cells were collected in DMEM medium for subsequent sequencing. Representative sorting plots illustrating the isolation strategy are shown in Supplementary Figure S2. Each retina was dissociated, stained, and sorted individually. However, CD45^+^ and/or CD11b^+^ cells recovered from the four retinas belonging to the same experimental group were collected into a common tube prior to library preparation, generating one scRNA-seq library for the control group and one scRNA-seq library for the xenotransplanted group. Therefore, the scRNA-seq dataset represents pooled biological material from four retinas per condition rather than individual biological replicates.

Single-cell RNA-seq libraries were prepared at the Servicio de Biología Molecular-ACTI of the Universidad de Murcia (Murcia, Spain) from the cell suspension using the Chromium Next GEM Single Cell 3′ Reagent Kits v3.1 (Dual Index) Kit (10× Genomics; CG000315, Bonsai Lab, Madrid, Spain) according to the manufacturer’s instructions. The expected cell recovery ranged between 1000 and 8000 target cells. Libraries were sequenced on an Illumina NovaSeq platform (Novogene Ltd., Cambridge, UK).

### 2.6. Single-Cell RNA-Seq Analysis

Raw FASTQ files were processed using Cell Ranger (v3.1) to generate gene-cell count matrices. Following FACS isolation, 2395 CD45^+^/CD11b^+^ cells were recovered from pooled control retinas and 16,665 cells from pooled xenotransplanted retinas. Before quality control, 181 and 1604 cells, respectively, were retained in the sequencing dataset. After quality filtering and doublet removal, 109 control cells and 1122 xenotransplanted cells were used for downstream analyses.

Downstream analysis was performed in R (v4.2) using the Seurat package (v4.0). Briefly, individual Seurat objects were created for the control and xenotransplanted samples, which were then merged into a single dataset. Both libraries were prepared and sequenced simultaneously using the same experimental workflow; therefore, no batch-correction procedure was applied prior to integration.

Quality metrics, including the number of detected genes, total UMI counts, and percentage of mitochondrial reads, were computed and used to exclude cells with fewer than 200 genes, more than 4000 genes, or over 10% mitochondrial content. Putative doublets were identified and removed using the DoubletFinder package over the first ten principal components with an optimized pK value. Data normalization was carried out using the SCTransform function, and the top 2000 highly variable features were identified using the FindVariableFeatures function. The dataset was then scaled via the ScaleData function while regressing out both feature count and mitochondrial percentage. Graph-based clustering was performed using the Louvain algorithm implemented in Seurat (FindClusters function) with a resolution parameter of 0.5 and the first 20 principal components as input. Uniform Manifold Approximation and Projection (UMAP) embeddings were generated with the RunUMAP function using the first 20 PCs. Cell-type annotation was performed using differential expression analysis together with canonical positive and negative marker genes. Annotated populations were assigned based on the combined expression of multiple lineage-specific markers rather than on single-marker expression. Cluster-specific marker genes were detected with the FindAllMarkers function, and differential expression analysis between the different populations was performed using the FindMarkers function (Seurat v4.0) with the default Wilcoxon rank-sum test and thresholds of |log_2_ fold change| > 0.322 and Bonferroni-adjusted *p* < 0.05.

Because one pooled library was generated per experimental condition, differential expression analyses and cell-type abundance estimates were performed on pooled single-cell data rather than at the biological replicate level. Consequently, cell-type proportions were calculated directly from the pooled cells within each condition and should be interpreted as descriptive estimates rather than biological replicate-based comparisons. Accordingly, cell-type proportions were used descriptively and were not subjected to formal differential abundance testing at the biological replicate level.

Functional enrichment of these differentially expressed genes was assessed using ReactomeGSA and g:Profiler. All figures, including violin plots, UMAPs, volcano plots, and heatmaps, were generated in R with ggplot2, cowplot, and patchwork.

### 2.7. Flow Cytometry of Lymphoid and Myeloid Immune Cells in the Retina and Spleen

Control (*n* = 5) or xenotransplanted (*n* = 5) retinas and spleens were dissected and immediately collected in Neurobasal medium supplemented with 10% FBS, 2% B-27, and 1% GlutaMAX™ for retinas, or in DMEM medium supplemented with 10% FBS and 1% GlutaMAX™ for spleens. Mechanical dissection was performed using a scalpel, followed by gentle pipetting of the tissues up and down twice to facilitate cell dissociation. Subsequently, 0.2% collagenase-A in DMEM medium was added, and the mixture was incubated at 37 °C for 30 min. The resulting cell suspensions were filtered through a 70-μm Corning™ cell strainer and centrifuged at 600 *g* for 5 min. The cell pellet was then resuspended in DMEM and stained with fluorochrome-labeled primary antibodies specific for the markers CD3 (clone 145-2C11; Invitrogen, Waltham, MA, USA), CD19 (clone 1D3; Tonbo Biosciences, San Diego, CA, USA), NK1.1 (clone PK136; Tonbo Biosciences, San Diego, CA, USA), CD11b (clone M1/70, Tonbo Biosciences, San Diego, CA, USA), Ly-6C (clone Monts 1; Elabscience, Houston, TX, USA) and Ly-6G (clone 1A8; Elabscience, Houston, TX, USA), along with their respective isotype controls.

Cell populations were gated based on FSC and SSC parameters, followed by viability discrimination using a fixable viability dye (Zombie NIR, BioLegend, San Diego, CA, USA), to exclude dead cells from the analysis. Fluorescence thresholds were set using the corresponding isotype controls for each fluorochrome (Supplementary Figure S3A,B). After a 30-min incubation at 4 °C, cells were washed twice and analyzed using a BD LSRFortessaTM flow cytometer (Becton Dickinson, Franklin Lakes, NJ, USA). Data were analyzed using Kaluza (London, UK) and FlowJo v10.10.0 (Ashland, OR, USA) software at the Tissue Culture Facility (ACTI, University of Murcia and IMIB).

### 2.8. Retinal Cross Sections and Image Acquisition

For immunohistofluorescence experiments on retinal cross-sections, animals (*n* = 5 control retinas, 5 xenotransplanted retinas) were euthanised with pentobarbital and immediately perfused transcardially with 0.9% saline solution, followed by 4% paraformaldehyde in 0.1 M phosphate buffer. Enucleated eyes were sequentially submerged in increasing concentrations of sucrose, embedded in Tissue-Tek^®^ O.C.T. compound (Sakura-Finetek, Barcelona, Spain) and stored at −80 °C until sectioning. Cryosections were cut at 16-µm thickness as previously described [31].

Immunodetection was performed as previously described [31,32]. Briefly, sections were washed three times with PBS with 0.1% Triton X-100 (Merck Life Science, Madrid, Spain) and incubated overnight at 4 °C with primary antibodies diluted in PBS containing 2% Triton X-100 and 2% Normal Donkey Serum or 2% Normal Goat Serum (Jackson ImmunoResearch Europe Ltd., Cambridge, UK). Primary antibodies were: rat anti-CD3 (1:1000 dilution; clone KT3; Abcam, Cambridge, UK), rabbit anti-CD19 (1:500 dilution; clone EPR23174-145, Abcam, Cambridge, UK), Alexa Fluor 488 rat anti-Ly-6G (1:400 dilution; clone 1A8, BioLegend, San Diego, CA, USA), rat anti-Ly-6C (1:500 dilution; clone ER-MP20, Santa Cruz Biotechnology, Dallas, TX, USA), rat anti-NKp26 (1:200 dilution, clone 29A1.4,BioLegend, San Diego, CA, USA), rat anti-CD45.2 (1:500 dilution, clone 104, eBiosciences, San Diego, CA, USA), rabbit anti-Iba1 (1:500 dilution; clone EPR16588, Abcam, Cambridge, UK), and guinea pig anti-Iba1 (1/500 dilution, clone Gp311H9, Synaptic Systems GmbH, Goettingen, Germany).

Secondary detection was performed using Alexa Fluor-conjugated secondary antibodies (1:500; Molecular Probes, ThermoFisher Scientific, Madrid, Spain) for all primary antibodies, except for the Ly-6G antibody, which was directly fluorochrome-labeled.

Finally, cross-sections were mounted using an anti-fading medium containing DAPI to counterstain cell nuclei (H-1200, Vectashield^®^, Vector Laboratories Inc., Burlingame, CA, USA). Images were acquired using a Leica DM6B epifluorescence microscope (Leica, Wetzlar, Germany).

### 2.9. Detection of Antibodies Against hBM-MSC Surface Antigens

#### 2.9.1. Plasma Collection

Blood was collected by intracardial puncture from control (*n* = 4) or xenotransplanted (*n* = 8) mice euthanised with an overdose of pentobarbital. Samples were immediately mixed with a citrate buffer solution (3.3% in double-distilled water) and kept on ice for 15 min. The blood was then centrifuged at 3000 rpm for 10 min at 4 °C. The resulting plasma was collected and immediately frozen at −80 °C until further analysis.

#### 2.9.2. Flow Cytometry

Plasma samples from control or xenotransplanted mice were incubated overnight at 4 °C with cultured human or murine (negative control) BM-MSCs. Briefly, BM-MSCs were seeded onto glass coverslips pre-coated with 0.1% gelatine solution in PBS (Sigma-Aldrich, Madrid, Spain) at a density of 2.5 × 10^4^ cells/mm^2^ and cultured for two days at 37 °C to allow cell adhesion. After this, the cells were incubated overnight at 4 °C with plasma from control or xenotransplanted mice, having first been treated with a human FcR blocking reagent (Miltenyi Biotec, Bergisch Gladback, Germany). After two washes with PBS, cells were incubated with phycoerythrin-labelled anti-mouse IgG (H + L) or Dyelight 488-labelled anti-mouse IgM antibodies (1:500; Molecular Probes, Thermo Fisher Scientific, Madrid, Spain) and analysed by flow cytometry as previously described.

#### 2.9.3. Immunodetection

Human or mouse BM-MSCs seeded as above were subsequently incubated with plasma from control or xenotransplanted mice overnight at 4 °C. The following day, cells were rinsed twice with PBS, incubated with Alexa Fluor 488-labelled anti-mouse IgG (H + L) or anti-mouse IgM antibodies (1:500; Molecular Probes, Thermo Fisher Scientific, Madrid, Spain), and finally mounted using an anti-fading medium containing DAPI for nuclear counterstaining. Samples were imaged using the same epifluorescence microscopy setup as previously described.

### 2.10. Statistics

Data analyses were not blinded. Data were analysed and plotted using GraphPad Prism v.7 (GraphPad Software, San Diego, CA, USA). Results are presented as scatter dot plots displaying individual data points along with the mean ± standard deviation (SD). Statistical analyses were performed using the non-parametric Mann–Whitney U test for comparison between control and xenotransplanted groups, as detailed in the corresponding figure legends. For scRNA-seq analyses, differential gene expression was performed using Seurat FindMarkers with the Wilcoxon rank-sum test and Bonferroni-adjusted *p*-values. Exact *p*-values are reported whenever possible, and differences were considered statistically significant at *p* < 0.05.

## 3. Results

### 3.1. Exploratory Single-Cell Transcriptomic Profile Identifies Candidate Immune Cell Populations Associated with the Xenogeneic Retinal Immune Response

Previously, we observed that in retinas xenotransplanted with human BM-MSCs, there was infiltration of CD45^+^/Iba1^−^ cells (Supplementary Figure S4A and [23]). These cells, likely leukocytes, were detected not only in the vitreous but also within the retinal parenchyma. Within this milieu, there were also CD45^+^/Iba1^+^ cells, which likely represented activated microglia or infiltrated macrophages, as well as CD45^−^/Iba1^+^ cells, which were consistent with resting or early-stage activated microglia/macrophages.

To obtain an initial transcriptomic overview of the cellular diversity and transcriptional profiles of these immune populations, we performed exploratory scRNA-seq analyses on CD11b^+^ and/or CD45^+^ cells isolated from control and xenotransplanted retinas. CD11b was chosen instead of Iba1 as a marker for identifying microglia/macrophages due to its surface expression, which facilitates the selection and isolation of viable cells by fluorescence-activated cell sorting (FACS). Consistent with the expected biological differences between healthy and xenotransplanted retinas, substantially more CD45^+^/CD11b^+^ cells were recovered from xenotransplanted animals than from controls (2395 cells were obtained from pooled control retinas and 16,665 cells from pooled xenotransplanted retinas). This difference was expected, as healthy retinas contain very few CD45^+^/CD11b^+^ immune cells, whereas xenogeneic BM-MSC transplantation promotes immune cell recruitment and activation.

**Figure 2:**
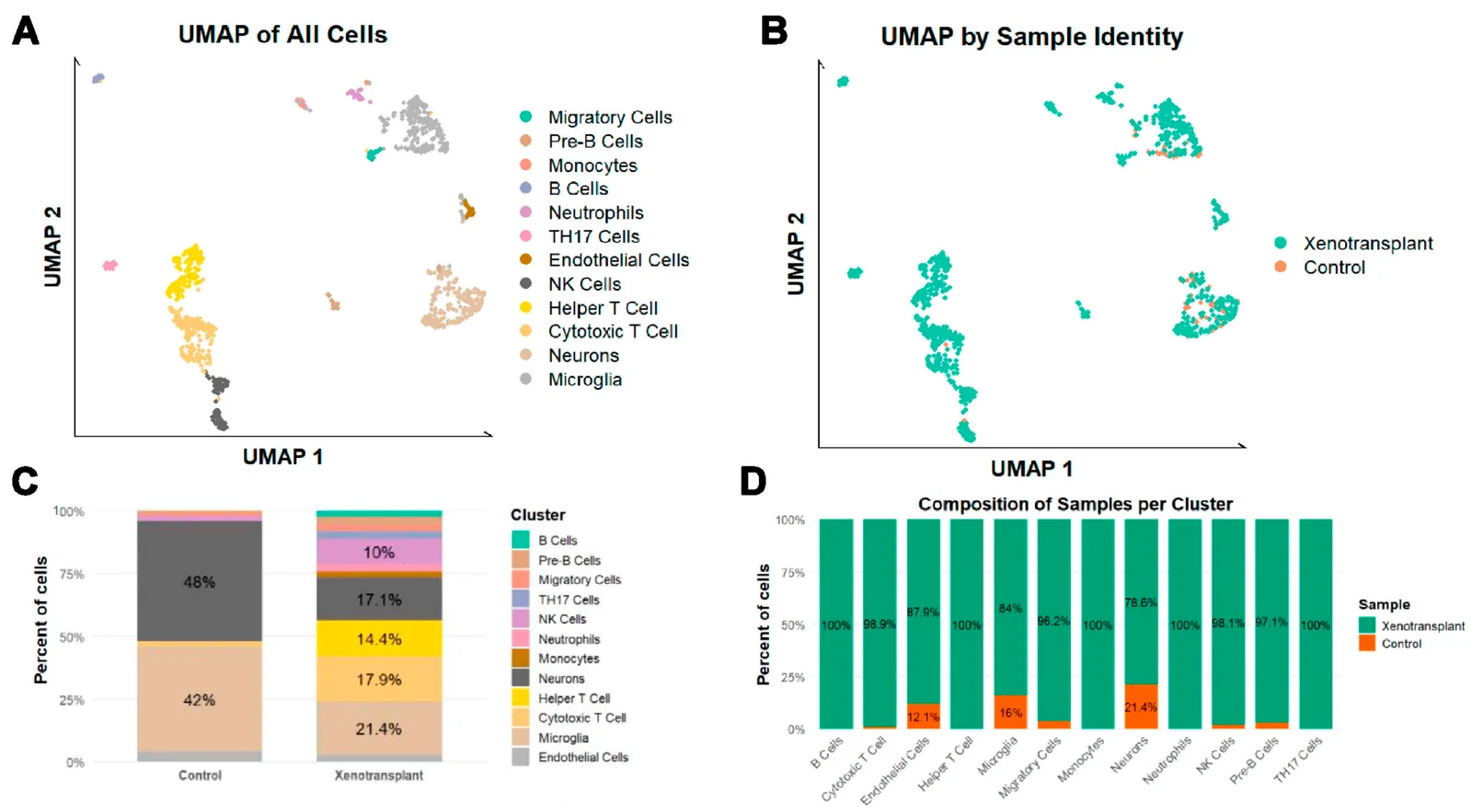
Myeloid and lymphoid clusters appear in the retina after xenotransplantation. (**A**) UMAP of single-cell transcriptomes identifying major retinal cell types. Each dot represents an individual cell plotted in the UMAP embedding based on gene expression profile. Cells are coloured according to assigned cell types inferred from canonical marker gene expression. The x- and y-axes represent UMAP dimensions 1 and 2, respectively. This analysis reveals transcriptionally distinct populations, including T cells, B cells, monocytes, and NK cells. (**B**) UMAP of cellular clusters coloured by sample origin: xenotransplanted animals (green) and control animals (orange). (**C**) Bar graphs showing the percentage composition of each identified cell population in control (left) and xenotransplanted (right) retinas. (**D**) Comparative bar graphs illustrating the relative distribution (percentage) of each cell population between control and xenotransplanted groups.

**Figure 3:**
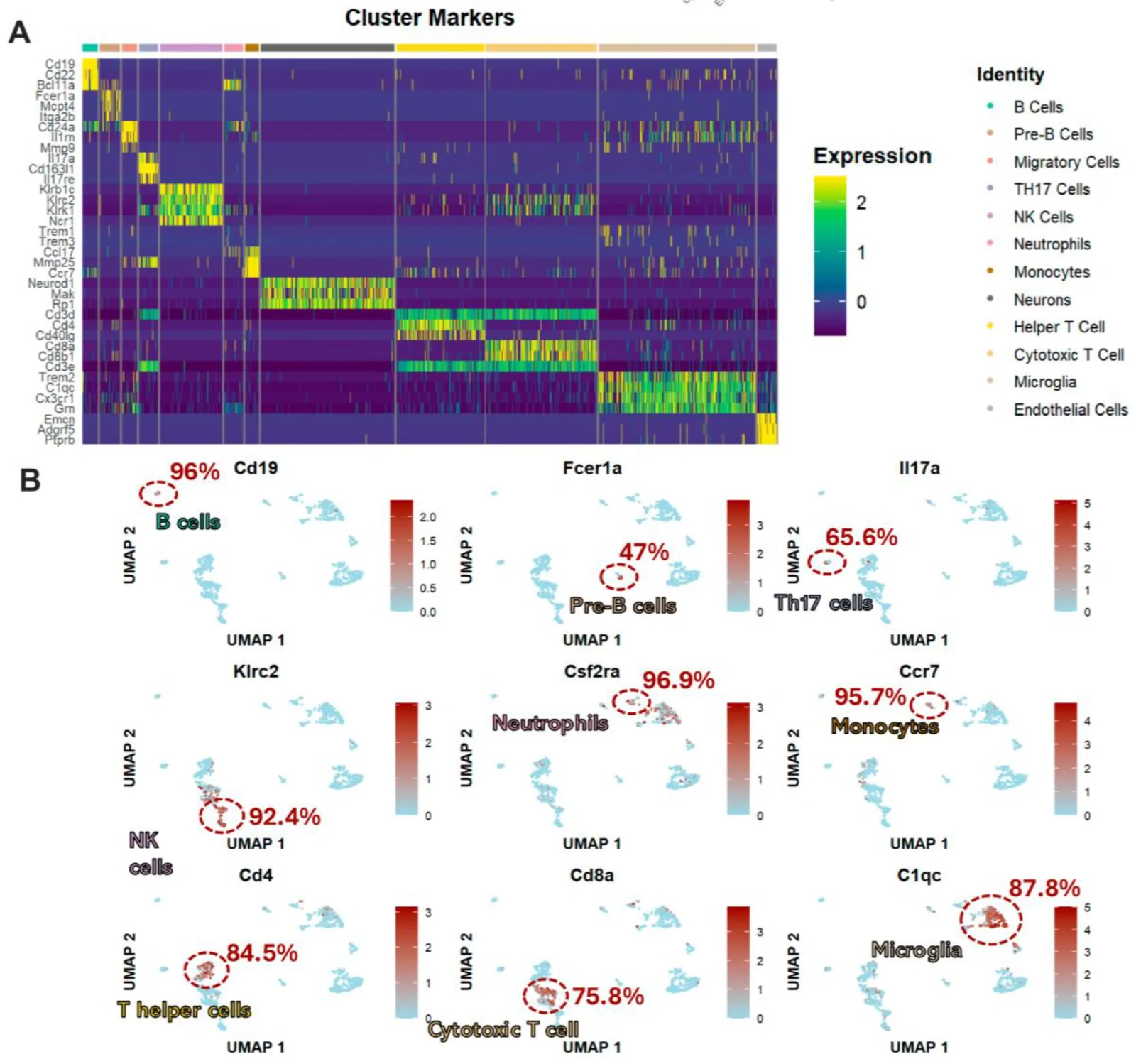
Annotation of cell clusters based on cluster-specific marker genes. (**A**) Visualization of cluster-specific marker genes from single-cell RNA sequencing. Expression levels are represented by colour intensity and grouped according to cell type identity. (**B**) UMAP highlighting subclusters of identified immune cell types, coloured based on the expression of specific marker genes. Relative marker expression levels within each subcluster are indicated as percentages.

After preprocessing with Cell Ranger and downstream analysis with Seurat, UMAP dimensionality reduction revealed 12 transcriptionally distinct cellular clusters (Figure 2A,B). Single-cell transcriptomic profiling revealed a heterogeneous cellular landscape comprising transcriptionally distinct clusters in control versus xenotransplanted retinal samples (Figure 2C,D and Figure 3A,B). These results show the diversity of immune cell populations and their associated functions in the retina. Notably, descriptive analysis of the pooled dataset revealed an apparent enrichment of cytotoxic T cells (17.9%), helper T cells (14.4%) and NK cells (10%) in xenotransplanted retinas compared to controls (Figure 2C). Because one pooled library was generated per experimental condition, these proportions should be interpreted as descriptive observations rather than formal biological replicate-based comparisons. Vehicle-injected controls were not included because previous work showed that vehicle injection did not reproduce the leukocyte infiltration or microglial changes induced by xenogeneic BM-MSC transplantation [23]; therefore, intact animals served as physiological controls.

UMAP clustering revealed 12 distinct cell populations, including B cells, T cell subtypes (Th17, helper, and cytotoxic), monocytes, neutrophils, and microglia. Cell-type annotation was validated by canonical marker expression, such as *Cd19* in B cells (96%), *Il17a* in Th17 cells (65.6%), and *C1qc* in microglia (87.8%) (Figure 3B). Cluster annotation was further supported by the combined expression of positive and negative lineage markers (Supplementary Data File S1). Microglia expressed *Tmem119*, *P2ry12*, *Sall1* and *Fcrls* while lacking lymphoid-associated markers, whereas infiltrating myeloid cells expressed *Ccr7*, *Fscn1*, *Mmp25*, *Ccl17* and *Il12b*. Similarly, cytotoxic T cells and NK cells were distinguished by their respective T-cell and NK-cell transcriptional signatures, and the IL-17-producing T-cell population was annotated based on the combined expression of *Il17a*, *Il17re*, *Trdc*, *Tcrg-C1* and *Trdv4*.

**Figure 4:**
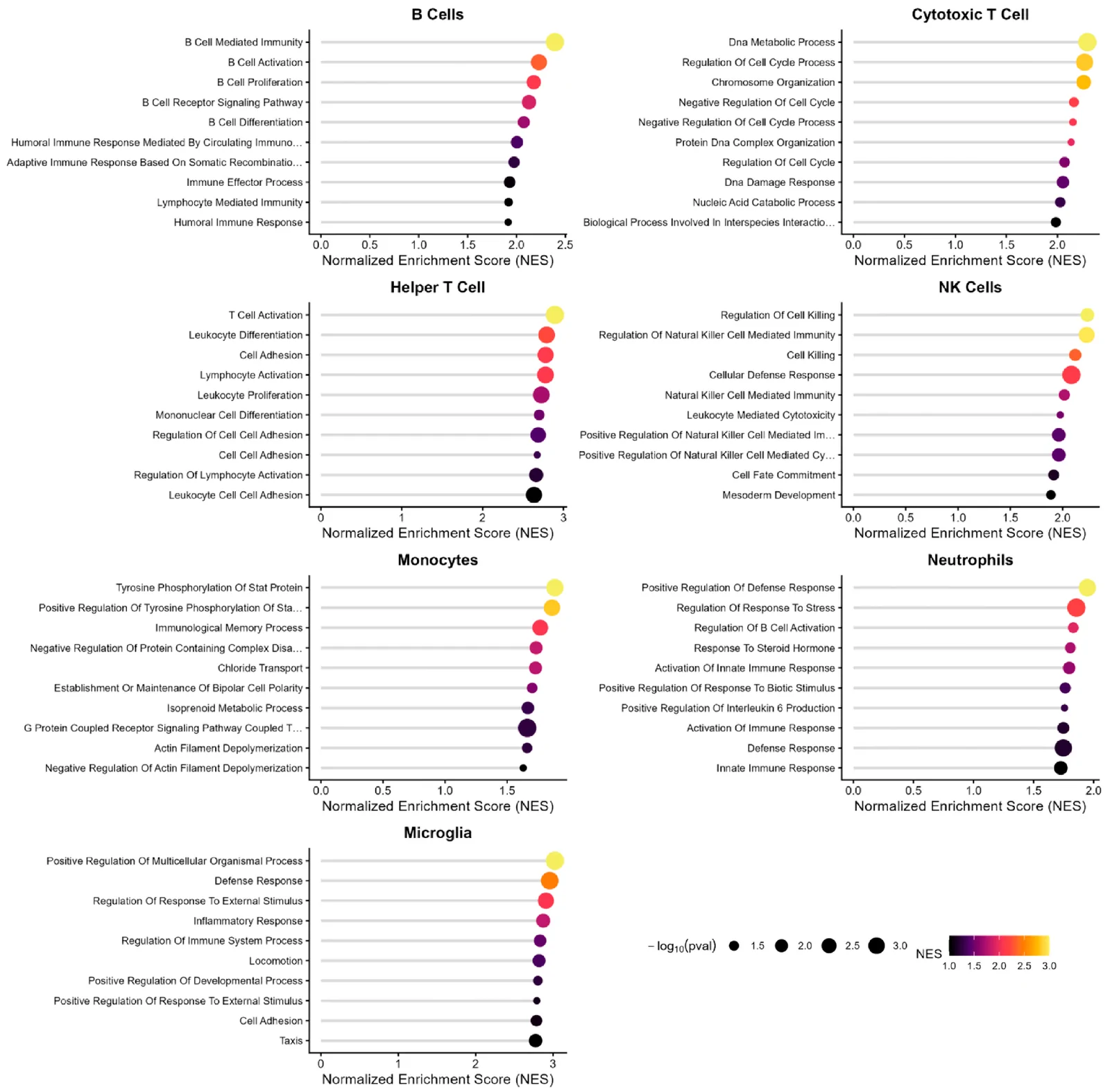
Top 10 enriched GO:BP pathways per immune cell type. Graphs showing the top ten most significantly enriched biological pathways for each cluster. Circle size represents the number of genes contributing to each pathway, while colour intensity reflects the adjusted *p*-value of enrichment. Bubble plot displaying the top hallmark pathways significantly upregulated in each cell cluster. Circle size and colour intensity correspond to the same parameters described in Figure 4.

Pathway enrichment analysis of the identified immune cell clusters revealed distinct activation signatures across cell types (Figure 4). The pooled xenotransplanted dataset exhibited enrichment of inflammatory and immune response pathways, notably those associated with adaptive immunity, including B cell activation, proliferation and differentiation, BCR signaling, and immunoglobulin production. Cytotoxic and helper T cells were enriched in pathways related to activation, proliferation, differentiation and cell adhesion, consistent with their effector functions. NK cells displayed enrichment for cytotoxicity and regulation of immune effector processes, whereas neutrophil-associated pathways involved activation, degranulation, and innate immune responses. Microglia and monocytes predominantly exhibited enrichment in pathways related to antigen processing and presentation, phagocytosis, and regulation of inflammatory responses, consistent with a potential role in initiating and sustaining innate immune activation in the context of xenotransplantation.

**Figure 5:**
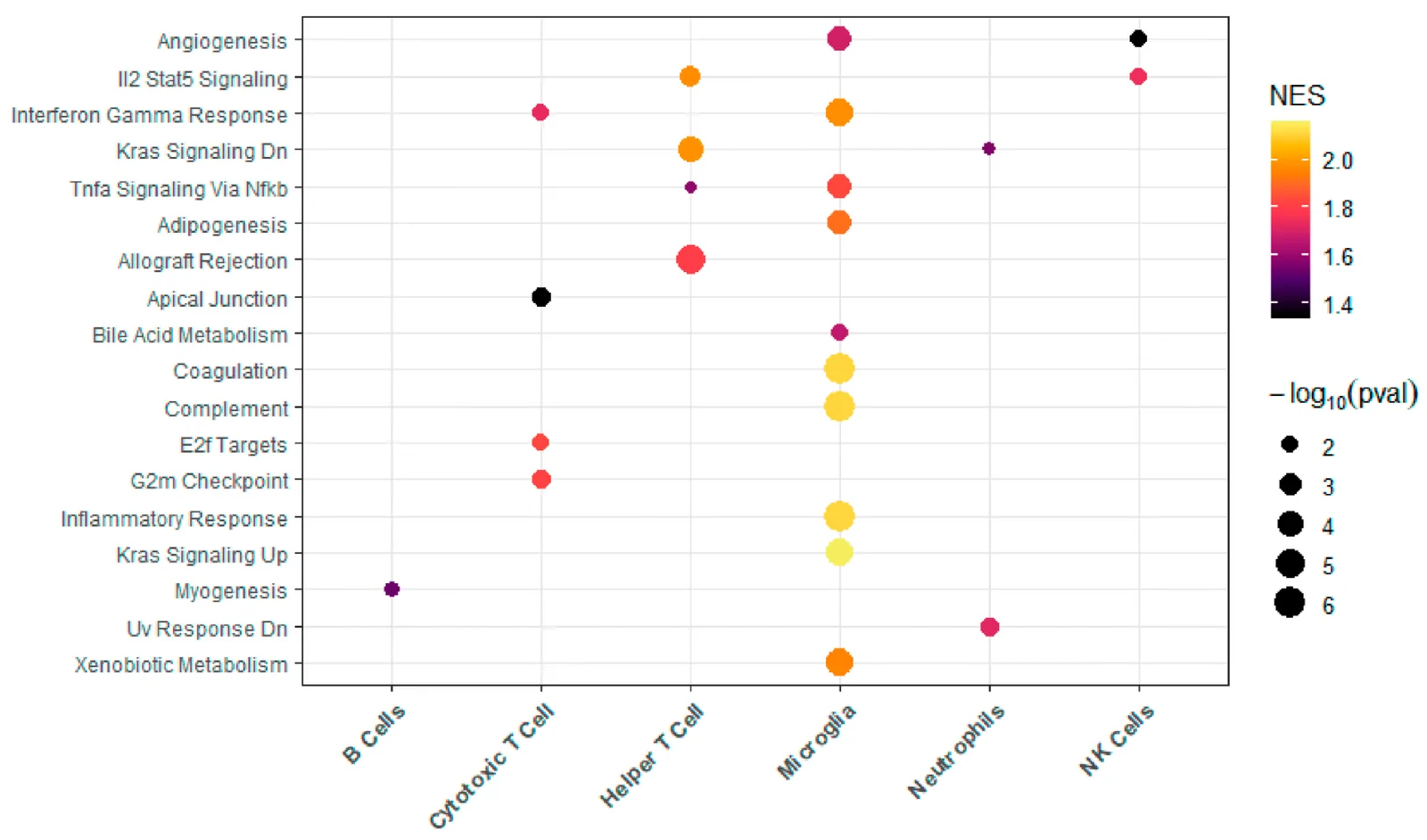
Top Hallmark pathways per immune population. Bubble plot displaying the top hallmark pathways significantly upregulated in each cell cluster. Circle size and colour intensity correspond to the same parameters described in Figure 4.

Top hallmark pathway analysis across immune population further corroborated these findings, revealing significant enrichment of inflammatory and immune-related signatures in xenotransplanted samples compared to controls (Figure 5). Prominent pathways included IFNγ response, TNF signalling via NF-κB, IL-2/STAT5 signalling, complement activation, and graft rejection-associated processes. Because control retinas yielded markedly fewer immune cells than xenotransplanted retinas, the representation of rare immune populations was reduced in the control dataset. This limitation should be considered when interpreting clustering results and downstream enrichment analyses.

Collectively, these exploratory transcriptomic findings are consistent with a coordinated activation of innate and adaptive immune-related pathways and were subsequently examined using independent validation approaches.

### 3.2. Leukocyte Infiltration in Retinas Following Xenogeneic Transplantation

To gain a deeper understanding of the immune response elicited by intravitreal administration of hBM-MSCs, we performed immunofluorescence staining on retinal cryosections, followed by flow cytometry analysis of both retinas and spleens obtained from the same control and xenotransplanted animals.

**Figure 6:**
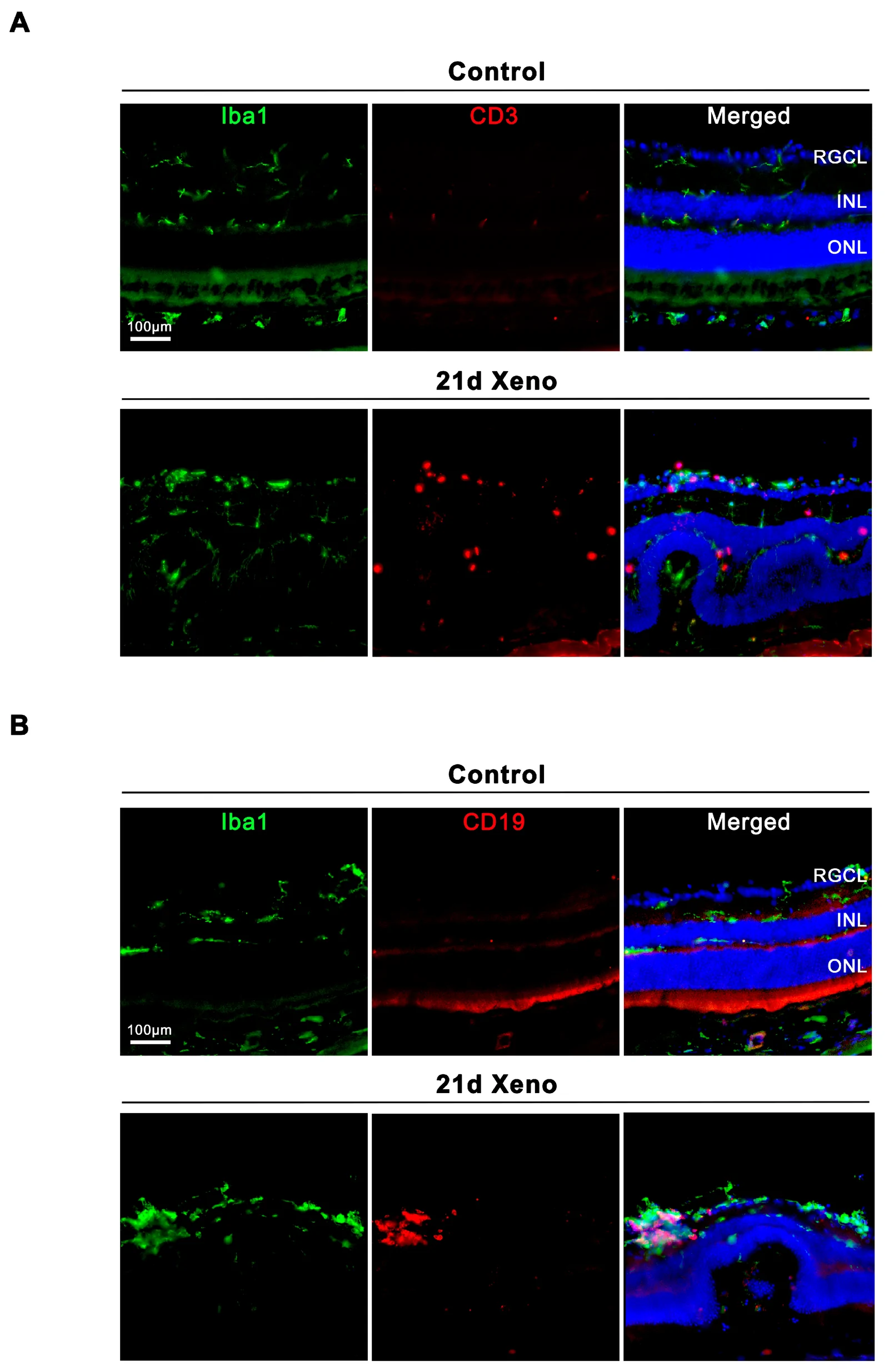
T and B cells infiltrate the xenotransplanted mouse retina. (**A**) Microscopy images showing the immunodetection of Iba1^+^ (green: microglia/macrophages) and CD3^+^ (red: T cells) cells in retinal sections from xenotransplanted and control animals. The right-hand column displays merged images counterstained with DAPI (blue) to visualize cell nuclei. (**B**) Microscopy images showing the immunodetection of Iba1^+^ cells (green: microglia/macrophages) and CD19^+^ cells (red: B cells) in retinal sections from xenotransplanted and control animals. The right column shows the merged image counterstained with DAPI (blue) to visualize cell nuclei. Abbreviations: RGCL: retinal ganglion cell layer; INL: inner nuclear layer; ONL: outer nuclear layer; Xeno: xenotrasplanted animals.

**Figure 7:**
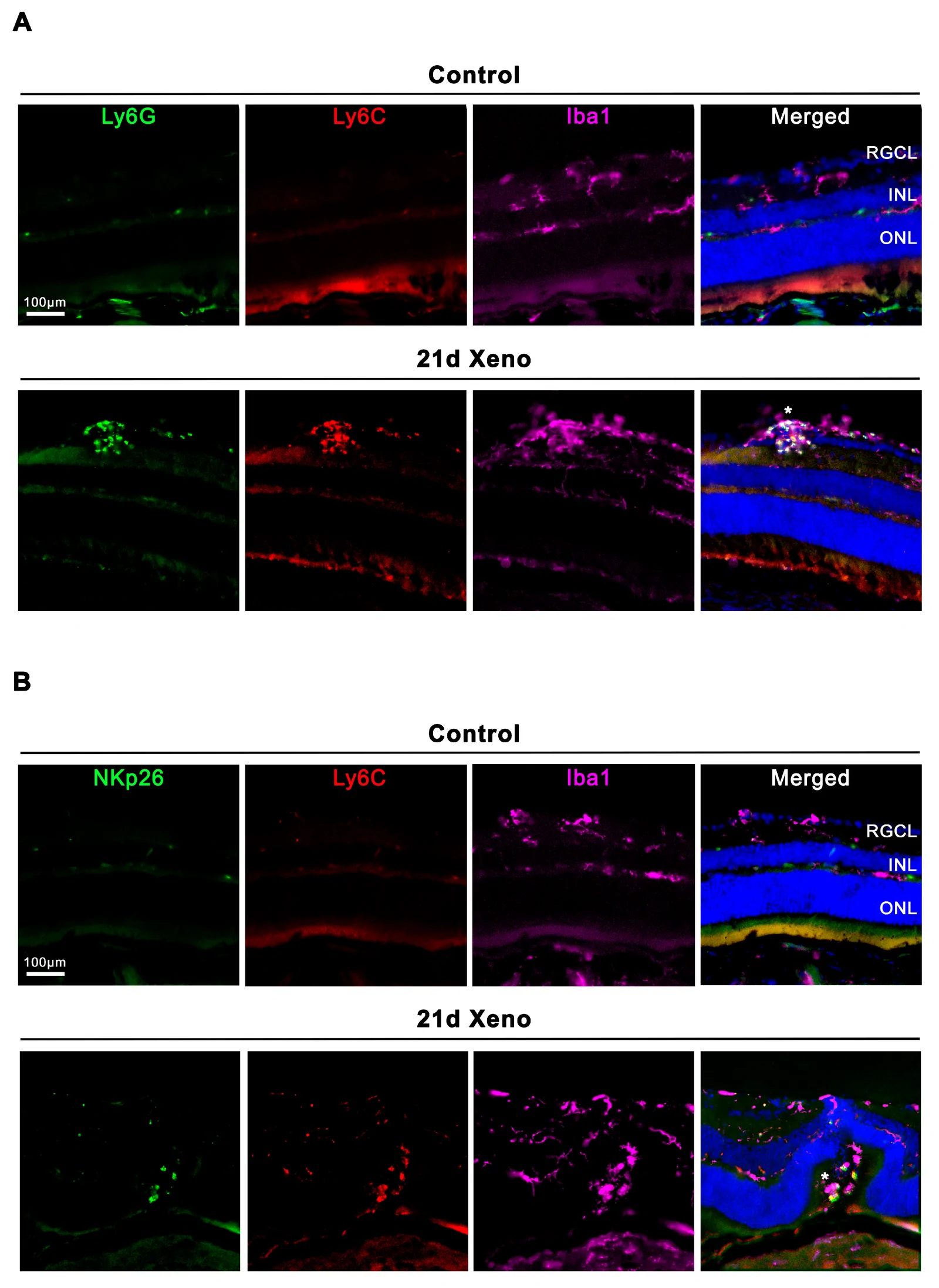
Neutrophil and NK cell infiltrate the xenotransplanted mouse retina. (**A**) Microscopy images showing the immunodetection of Ly-6G^+^ (green) Ly-6C^+^ (red), and Iba1^+^ (purple) cells in retinal sections from xenotransplanted and control animals. Asterisks mark Ly-6G^+^ Ly-6C^+^ Iba1^+^ cells (monocytes). The right column shows the merged image counterstained with DAPI (blue) to visualize cell nuclei. (**B**) Microscopy images showing the immunodetection of NKp26^+^ (green) Ly-6C^+^ (red), and Iba1^+^ (purple) cells in retinal sections from xenotransplanted and control animals. Asterisks mark NK cell population (NKp26^+^ Ly-6C^+^ Iba1^−^). The right column shows the merged image counterstained with DAPI (blue) to visualize cell nuclei. Abbreviations: RGCL: retinal ganglion cell layer; INL: inner nuclear layer; ONL: outer nuclear layer; Xeno: xenotrasplanted animals.

We observed increased levels of infiltrating CD3^+^ T cells, CD19^+^ B cells, Ly-6G^+^ neutrophils, Ly-6C^+^ monocytes, and NKp26^+^ NK cells in the retinas of xenotransplanted animals compared to controls (Figure 6 and Figure 7). Notably, adaptive immune cells, particularly T and B lymphocytes, were predominantly localized around the retinal folds formed following xenogeneic transplantation of hBM-MSCs. Within these regions, CD3^+^ T cells were more abundant than CD19^+^ B cells (Figure 6). NKp26^+^ NK cells were also identified in close proximity to the retinal foldings, primarily localized within the outer retinal layers (Figure 7), suggesting a potential route of infiltration through the choroid.

**Figure 8:**
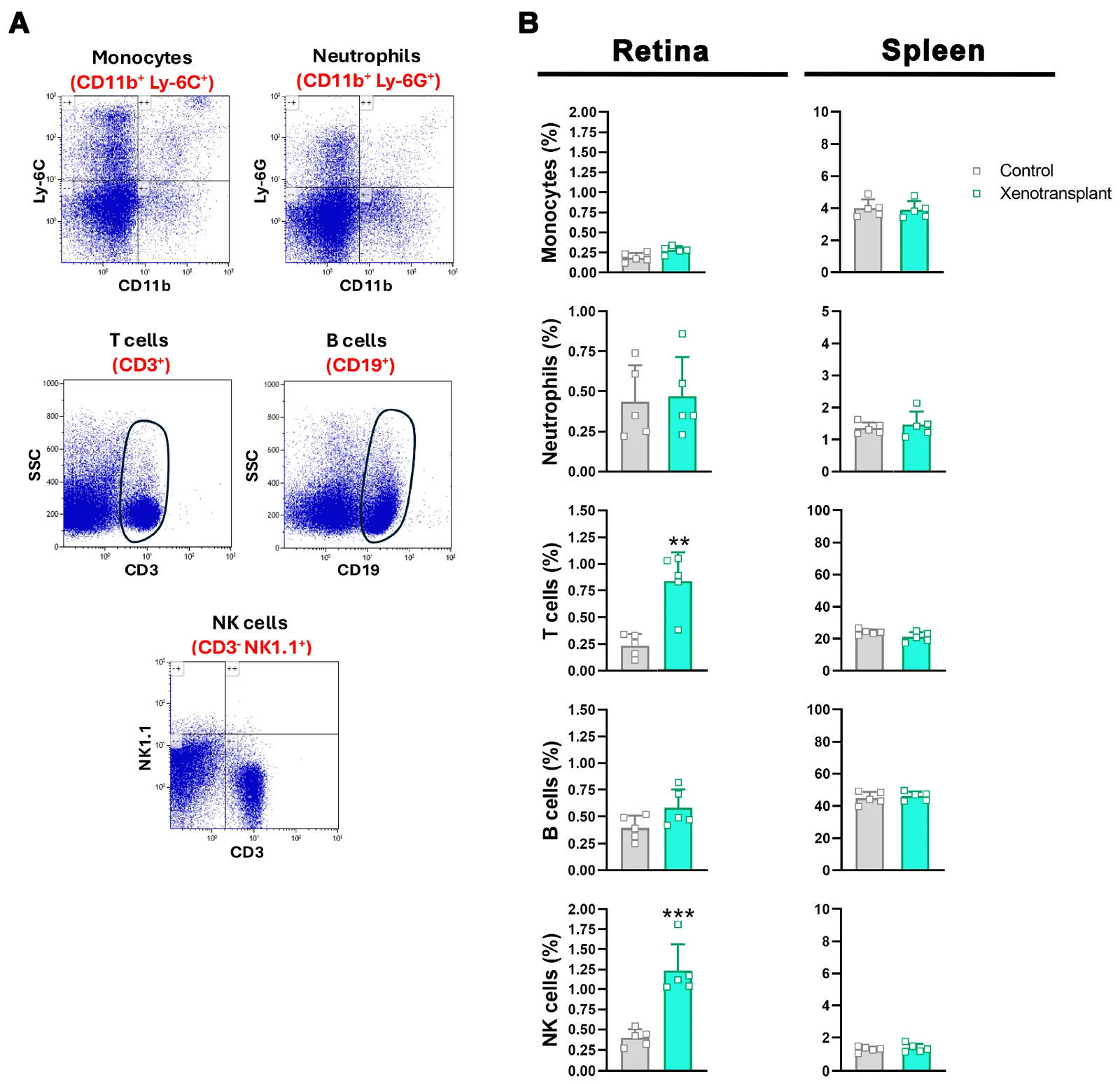
Immune cell populations in the retina and spleen. (**A**) Representative dot plots illustrating the identification of immune cell populations based on marker expression, including monocytes (CD11b^+^ Ly-6C^+^), neutrophils (CD11b^+^ Ly-6G^+^), T cells (CD3^+^), B (CD19^+^), and NK (CD3^−^ NK1.1^+^) cells by flow cytometry. (**B**) Quantification of immune cell populations in retina and spleen samples from control and xenotransplanted animals. Bar graphs represent mean percentage ± SD in both retina and spleen samples from control and xenotrasplanted animals. *n* = 5 retinas and spleens per group. ** *p* = 0.008 and *** *p* = 0.0006, according to the non-parametric Mann–Whitney U test.

Flow cytometry analysis of dissociated retinal cells revealed increased immune cell infiltration in xenotransplanted retinas, particularly CD3^+^ T cells and NK1.1^+^ NK cells (*p* = 0.008 and *p* = 0.0006, respectively; Figure 8A,B), supporting the recruitment of both innate and adaptive immune cells. In contrast, no significant differences were observed in splenic immune cell populations, suggesting that the immune response was primarily localized to the site of xenotransplantation (Figure 8B). Notably, the immune cell subsets identified by flow cytometry in the retina closely matched those detected by scRNA-seq analysis, as shown in Figure 3B.

### 3.3. Antibodies Against Human BM-MSCs Are Detected in the Xenotransplanted Animals

**Figure 9:**
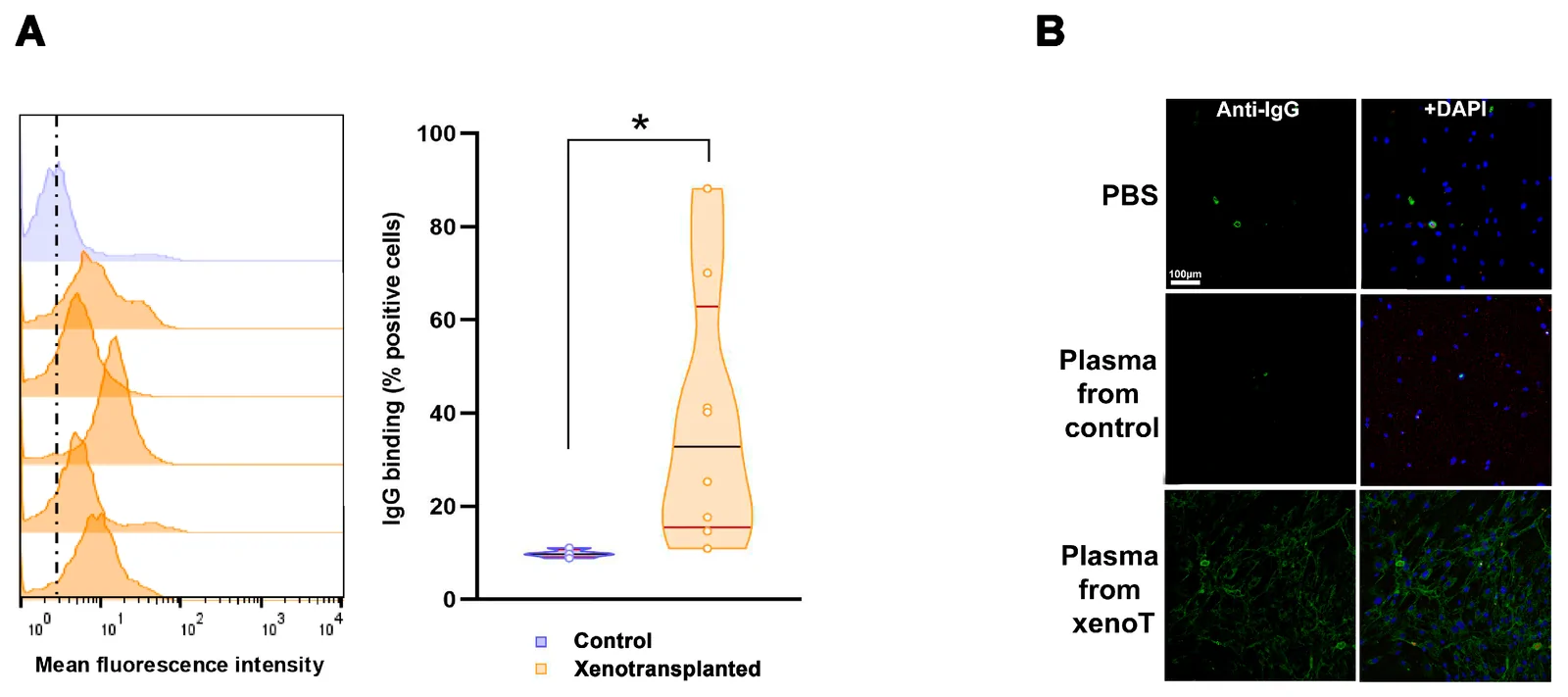
Antibodies against human cells in xenotransplanted mice. (**A**) From left to right: histograms showing the percentage of IgG-bound hBM-MSCs after incubation with serum from control animals (purple) and xenotransplanted animals (orange), and violin plots showing the percentage of IgG-bound hBM-MSCs after incubation with serum from control animals (purple; *n* = 4) and xenotransplanted animals (orange; *n* = 8 independent samples). * *p* = 0.02, according to the non-parametric Mann–Whitney U test. (**B**) Representative images of fixed hBM-MSCs incubated with serum from xenotransplanted and control animals, followed by detection with a fluorescent-conjugated anti-mouse Ig (H + L) secondary antibody (green, left column). The middle column shows control staining using only the secondary antibody (no primary serum). The right column displays merged images counterstained with DAPI to visualize cell nuclei. Abbreviations: XenoT: Xenotransplanted animals.

To assess the presence of circulating antibodies directed against hBM-MSCs, plasma samples from xenotransplanted and control mice were incubated in vitro with cultured hBM-MSCs. The cells were subsequently analyzed by flow cytometry (Figure 9A) or fixed for immunodetection (Figure 9B), using fluorescently labelled anti-mouse IgG (H + L) and IgM antibodies.

Flow cytometry assays showed a significant increase in fluorescence intensity when hBM-MSCs were incubated with plasma from xenotransplanted animals compared to plasma from controls. Quantitative analysis showed a significantly higher percentage of hBM-MSCs labelled with anti-mouse IgG antibodies in the xenotransplanted group (Mean ± SD: 38.55 ± 27.80%) versus controls (Mean ± SD: 9.87 ± 0.92%; *p* = 0.02) (Figure 9A). Importantly, control experiments using murine BM-MSCs (mBM-MSCs) exposed to plasma from either xenotransplanted or control animals showed no detectable signal, indicating preferential recognition of antigens expressed by hBM-MSCs. However, the present study was not designed to determine whether these antibodies are specific to hBM-MSCs or recognize human antigens more broadly.

Consistent with these findings, immunodetection analyses revealed no significant labelling of hBM-MSCs exposed to plasma from control animals (Figure 9B), or when mBM-MSCs were used as targets. However, a clear signal was detected in hBM-MSCs incubated with plasma from xenotransplanted animals (Figure 9B). Notably, no significant IgM reactivity was detected by either flow cytometry (*p* = 0.11) or immunodetection analyses (Supplementary Figure S4B,C). Together, these results are consistent with the presence of an IgG-mediated humoral immune response directed against antigens expressed by intravitreally transplanted hBM-MSCs.

These findings should therefore not be generalized to all MSC therapies, CNS compartments, or transplantation settings. The intravitreal, MHC-mismatched human–mouse model limits clinical extrapolation, as the species mismatch likely contributed substantially to the observed response and may not reflect autologous, HLA-matched, or immunosuppressed transplantation.

## 4. Discussion

The present study investigated the immune response elicited by intravitreal xenogeneic transplantation of hBM-MSCs into the healthy mouse retina, a CNS model. Using exploratory single-cell transcriptomics complemented by immunohistochemistry, flow cytometry, and plasma antibody analyses, we identified evidence of both innate and adaptive immune activation following transplantation. The main findings include the infiltration of immune cells into the retina, the apparent enrichment of cytotoxic T cells, helper T cells, and NK cells, and the generation of antibodies against human antigens, collectively indicating that xenogeneic BM-MSC transplantation can overcome local immune regulatory mechanisms and trigger a robust host immune response.

Despite the immunoprivileged status of the eye [2] and the retina [3], our findings demonstrate that intravitreal administration of hBM-MSCs in mice can elicit a robust local immune response. Specifically, we detected the production of antibodies targeting human antigens and local leukocyte infiltration within the retina, whereas no significant systemic cellular immune activation was detected in the spleen. These results suggest that the immune reaction remains largely confined to the ocular xenotransplantation site yet is sufficient to compromise graft survival, with the retinal folds observed following hBM-MSC transplantation potentially contributing to local immune cell accumulation.

Exploratory single-cell transcriptomic analysis provided an initial overview of the immune landscape of this response, revealing distinct immune cell populations infiltrating the retina after intravitreal hBM-MSC xenotransplantation. The apparent enrichment of cytotoxic T cells, helper T cells, and NK cells in the pooled xenotransplanted transcriptomic dataset, together with the independent immunohistochemical and flow cytometric findings, is consistent with a prominent effector-cell response to the xenogeneic graft. Cytotoxic T cells, which were markedly enriched (17.9%), are likely key mediators of direct graft damage through perforin- and granzyme-dependent mechanisms, while helper T cells (14.4%) amplify this response by recruiting and activating additional immune cells and microglia via TCR signalling and cytokine secretion [33]. The presence of NK cells (10%) further points to an innate immune contribution, as these cells can recognize and lyse cells lacking self-MHC molecules without prior sensitization. Collectively, this effector-rich immune profile may overcome local retinal immune regulation and promote localized tissue inflammation [25]. A technical consideration is that scRNA-seq analysis of isolated CD45^+^/CD11b^+^ retinal cells also detected transcripts typically associated with neuronal and endothelial cells. These signals may reflect transcript contamination arising from persistent cell–cell aggregates or ambient RNA carryover during tissue dissociation and single-cell processing, phenomena known to affect droplet-based scRNA-seq datasets [34].

Our previous observations also highlight the role of microglia in ocular transplantation [23]. Intravitreally injected MSCs, whether xenogeneic, allogeneic, or syngeneic, induce microglial migration toward the epiretinal membrane they form, suggesting that this innate response may contribute to their limited survival within the CNS. Interestingly, syngeneic stem cells of neuroectodermal origin, which share developmental lineage with the CNS, also form an epiretinal membrane following intravitreal administration. However, unlike mesoderm-derived MSCs, these cells exhibit long-term survival without triggering retinal gliosis or microglial infiltration into the vitreous [13]. This differential host response to syngeneic transplantation suggests that graft immune rejection may depend not only on species compatibility but also on the developmental origin of the transplanted cells, an aspect rarely considered in preclinical models.

In addition to the cellular response, the detection of antibodies against human antigens suggests that humoral mechanisms may contribute to graft clearance and potentially influence responses to subsequent transplantation. Once antibodies bind to the surface of transplanted hBM-MSCs, multiple effector mechanisms can mediate their clearance. One such mechanism is antibody-dependent cellular cytotoxicity (ADCC), in which NK cells engage the Fc portion of bound IgG via FcγRIII (CD16), triggering perforin and granzyme release to induce apoptosis of the target cell [35]. Another is antibody-dependent cellular phagocytosis (ADCP), primarily mediated by macrophages and microglia, which engulf antibody-coated cells through Fc receptor interaction [36]. Additionally, the classical complement pathway can be activated by IgG or IgM bound to cell surfaces, leading to the formation of the membrane attack complex (MAC) and direct target cell lysis [37]. Together, these mechanisms represent plausible effector pathways that could contribute to reduced survival of xenogeneic hMSCs in the vitreous. However, their functional involvement was not directly tested in the present study and should therefore be interpreted as mechanistic hypotheses supported by the observed immune context.

Understanding these effector pathways opens opportunities for targeted immunomodulatory strategies. Future studies should explore local or transient immunosuppression, Fc engineering to reduce antibody effector functions, or encapsulation techniques to shield transplanted cells from immune recognition within the CNS, where immune privilege is often assumed but not guaranteed. Although immunosuppressive treatments have been shown to enhance xenograft survival and reduce host immune activation in experimental transplantation settings [4,38], they may also mask key immune mechanisms involved in graft recognition and rejection. Therefore, immunosuppression was intentionally not included in the present study, as our primary objective was to characterize the immune response elicited by xenogeneic hBM-MSC transplantation under physiological conditions. Importantly, the potential benefits and safety of these strategies should be specifically validated in the context of retinal xenotransplantation, as their impact on graft survival, retinal homeostasis, and local immune responses remains to be determined.

This study has some limitations. Sample sizes were modest, particularly for scRNA-seq, and samples were pooled without independent biological replicates. Consequently, observations regarding differential abundance, differential gene expression, and cell-type proportions could not account for between-animal variability or be statistically assessed at the biological replicate level. Therefore, the single-cell analyses should be interpreted as exploratory and hypothesis-generating. Importantly, the candidate immune-cell population and pathways identified by scRNA-seq were later explored using independent histological, flow cytometric, and antibody detection analyses, supporting the biological relevance of the observed immune response. The lower immune cell yield from control retinas may have limited the detection of rare populations and reduced the sensitivity of clustering and pathway analyses. Cell-type annotations were based on established transcriptional signatures and canonical markers but were not independently validated by lineage tracing or additional protein markers. Only male mice were studied, preventing assessment of sex-dependent responses. In addition, an allogeneic transplantation group was not included. While previous studies from our group showed that allogeneic murine BM-MSCs also elicit retinal immune activation [23], the xenogeneic model was intentionally selected to characterize host immune responses under conditions of maximal immune challenge. Future studies directly comparing allogeneic and xenogeneic transplantation would further enhance the translational relevance of these findings. Thus, the model provides a sensitive framework for identifying species-specific immune responses and for informing the design and interpretation of more translationally relevant preclinical studies.

## 5. Conclusions

The present study combined exploratory single-cell transcriptomics with flow cytometry, immunohistochemistry, and antibody profiling to characterize the immune response elicited by intravitreal xenogeneic hBM-MSC transplantation in the healthy retina. These complementary approaches provide convergent evidence of a coordinated immune response following intravitreal xenogeneic hBM-MSC transplantation, characterized by microglial activation, recruitment of peripheral leukocytes, and an IgG-mediated humoral response. Together, these findings advance our understanding of host-graft interactions in the retina, demonstrate that intravitreal xenogeneic cell transplantation can overcome local ocular and retinal immune regulation, and highlight immune rejection as a critical consideration for the safe and effective translation of stem cell-based therapies. Furthermore, they provide a valuable framework for the development of targeted immunomodulatory strategies and more translationally relevant preclinical models.

## Data Availability

The data supporting the findings of this study are available from the corresponding authors upon reasonable request. The scRNA-seq data has been deposited in the Gene Expression Omnibus (GEO) repository under accession number GSE337881.

## Supplementary Materials

The following supporting information can be downloaded at https://www.mdpi.com/article/10.3390/cells15211966/s1: Figure S1: Immunophenotypic and multilineage differentiation characterization of hBM-MSCs. (A) Flow cytometric analysis of hBM-MSCs showing expression of the mesenchymal markers CD73, CD90 and CD105, and lack of expression of the hematopoietic markers CD14, CD20, CD34 and CD45. Grey histograms correspond to isotype controls. (B) Representative images of adipogenic, osteogenic and chondrogenic differentiation. Adipogenic differentiation was assessed by Oil Red O staining, osteogenic differentiation by BCIP/NBT staining, and chondrogenic differentiation by Alcian blue/eosin staining. Scale bar: 200 μm. Figure S2: FACS strategy for CD45^+^/CD11b^+^ retinal immune cells isolation. Figure S3: Flow cytometry gating strategy. Figure S4: Leukocyte infiltration and IgM reactivity after xenogeneic hBM-MSC transplantation. Supplementary Data File S1: Complete differential expression analysis used for cluster annotation.

## Abbreviations

The following abbreviations are used in this manuscript:

hBM-MSCS	Human bone marrow mesenchymal stromal cells
BSA	Bovine serum albumin
CNS	Central nervous system
DMEM	Dulbecco’s modified eagle medium
FACS	Fluorescence-activated cell sorting
FBS	Fetal bovine serum
PBS	Phosphate-buffered saline
P/S	Penicillin/streptomycin
scRNA-seq	Single cell RNA sequencing
